# TeleWound Practice Within the Veterans Health Administration: Protocol for a Mixed Methods Program Evaluation

**DOI:** 10.2196/20139

**Published:** 2020-07-20

**Authors:** Bella Etingen, Jamie Patrianakos, Marissa Wirth, Timothy P Hogan, Bridget M Smith, Elizabeth Tarlov, Kevin T Stroupe, Rebecca Kartje, Frances M Weaver

**Affiliations:** 1 Center of Innovation for Complex Chronic Healthcare Edward Hines Jr Veterans Administration Hospital Hines, IL United States; 2 Center for Healthcare Organization and Implementation Research Edith Nourse Rogers Memorial Veterans Hospital Bedford, MA United States; 3 Department of Population and Data Sciences University of Texas Southwestern Medical Center Dallas, TX United States; 4 Northwestern University Feinberg School of Medicine Chicago, IL United States; 5 College of Nursing University of Illinois at Chicago Chicago, IL United States; 6 Parkinson School of Health Sciences and Public Health Loyola University Chicago Maywood, IL United States

**Keywords:** implementation, telehealth, wound care, veteran, protocol, wound, outcome

## Abstract

**Background:**

Chronic wounds, such as pressure injuries and diabetic foot ulcers, are a significant predictor of mortality. Veterans who reside in rural areas often have difficulty accessing care for their wounds. TeleWound Practice (TWP), a coordinated effort to incorporate telehealth into the provision of specialty care for patients with skin wounds, has the potential to increase access to wound care by allowing veterans to receive this care at nearby outpatient clinics or in their homes. The Veterans Health Administration (VA) is championing the rollout of the TWP, starting with regional implementation.

**Objective:**

This paper aims to describe the protocol for a mixed-methods program evaluation to assess the implementation and outcomes of TWP in VA.

**Methods:**

We are conducting a mixed-methods evaluation of 4 VA medical centers and their community-based outpatient clinics that are participating in the initial implementation of the TWP. Data will be collected from veterans, VA health care team members, and other key stakeholders (eg, clinical leadership). We will use qualitative methods (ie, semistructured interviews), site visits, and quantitative methods (ie, surveys, national VA administrative databases) to assess the process and reach of TWP implementation and its impact on veterans’ clinical outcomes and travel burdens and costs.

**Results:**

This program evaluation was funded in October 2019 as a Partnered Evaluation Initiative by the US Department of Veterans Affairs, Diffusion of Excellence Office, and Office of Research and Development, Health Services Research and Development Service, Quality Enhancement Research Initiative Program (PEC 19-310).

**Conclusions:**

Evaluation of the TWP will identify barriers and solutions to TeleWound implementation in a small number of sites that can be used to inform successful rollout of the TWP nationally. Our evaluation work will inform future efforts to scale up the TWP across VA and optimize reach of the program to veterans across the nation.

**International Registered Report Identifier (IRRID):**

DERR1-10.2196/20139

## Introduction

### Telehealth and Wound Care

Chronic skin wounds are a substantial health problem among veterans. Research has shown that within veteran cohorts, wounds are a more significant predictor of mortality than coronary artery disease, peripheral arterial disease, or stroke [1]. Veterans who reside in rural areas receive less specialty care services than their urban-dwelling counterparts [2,3] due to transportation-related and time-related barriers [4], indicating that there may be a gap between those who need wound care and those who receive it. As such, efforts are needed to improve access to specialty wound care services, particularly for veterans who reside in rural areas.

One strategy for improving access to care is to incorporate telehealth technologies into health care service delivery. Telehealth programs offered within the Veterans Health Administration (VA) have shown great promise in improving care delivery and outcomes among veterans. For example, receiving telehealth services has been associated with decreased health care use and inpatient length of stay; improvements in quality of life, satisfaction, and clinical outcomes; and reduced health care costs [5-8].

Specific to wound care, recent evidence supports the utility of incorporating telehealth into the delivery of wound care services, noting positive impacts such as reduced wound healing time, fewer adverse events, reduced travel costs, and enhanced ability of nursing staff to conduct comprehensive care [9-11]. The integration of telehealth and wound care may be particularly impactful within VA, where improving access to care, particularly access to specialty care services, for veterans living in rural and highly rural areas can be challenging. These challenges are due in large part to the fact that these veterans must often travel great distances to reach their nearest VA facility. Incorporating telehealth technology into wound care services provides a means to deliver care closer to (or even in) veterans’ homes. Within this context, VA’s TeleWound Practice (TWP) emerged.

### The Origins of VA’s TeleWound Practice

TWP encompasses a coordinated effort to incorporate telehealth technology into the delivery of specialty wound care services. This process can be asynchronous, such as taking a picture of a patient’s wound and sending it to a wound care specialist for review, referred to as store-and-forward telehealth (SFT), or synchronous, such as real-time video visits in which the patient is either in their home or a VA community-based outpatient clinic (CBOC) and has a video visit with a wound care specialist at a main VA hospital facility using clinical video telehealth (CVT) or VA Video Connect (VVC). Compared with traditional in-person clinic visits, TWP visits may result in decreased travel burden and cost, more flexible scheduling, and a greater likelihood of seeing the provider best matched to veterans’ needs. A potential advantage to the health care system is the more efficient use of scarce resources (eg, highly trained specialists). Additionally, TWP encompasses standardized care team member training and clinical documentation across the system, as well as an emphasis on interprofessional stakeholder collaboration.

Within VA, the TWP program originated at a VA medical center that serves a large number of veterans who live in rural or highly rural areas. The program was highly successful at this facility, decreasing patient travel burden related to wound care and eliciting high levels of patient satisfaction. Because of this early and encouraging success, the TWP was selected by VA for widescale rollout, beginning with regional-level implementation.

The regional implementation of TWP will leverage a multicomponent, facilitation-based implementation strategy. Components of the implementation strategy include (1) identifying a clinical champion at each facility to support local rollout of the TWP program; (2) facilitating recurring virtual meetings (akin to a learning collaborative) to engage key stakeholders, share lessons learned, and discuss strategies for overcoming barriers; and (3) deploying necessary training to staff. Through the program evaluation described here, VA national operations program offices have partnered with VA researchers to evaluate the TWP program implementation process and outcomes. Findings from this evaluation will be used to inform further rollout of the program nationally.

### Objective

This protocol represents the real-world application of evidence-based implementation science principles in a large learning health care system and the collaboration between operations and researchers to conduct program evaluations in an effort to improve widescale implementation of evidence-based interventions and in turn, improve patient care.

### Evaluation Aims

The proposed aims of our evaluation are to (1) evaluate the implementation process of the TWP program regional rollout, (2) assess the impact of the TWP on clinical outcomes related to wound care, and (3) assess the impact of the TWP on health care system outcomes related to wound care.

## Methods

### Conceptual Framework

To formulate our evaluation plan, we drew from a conceptual model frequently used in eHealth–related research and program evaluation, the Practical, Robust Implementation and Sustainability Model (PRISM) [12,13].

PRISM has 4 major domains: the intervention, the intervention recipients (eg, patients, clinical team members), the implementation and sustainability infrastructure, and the broader environment [12,13]. By using PRISM to guide our evaluation efforts, we will be able to account for the different stakeholders of the TWP, their perspectives on the intervention being implemented, and other characteristics that could influence implementation efforts.

PRISM outcome measures follow the Reach, Effectiveness, Adoption, Implementation, Maintenance (RE-AIM) framework [12,13]. RE-AIM evaluates implementation processes and outcomes along several dimensions, including reach, effectiveness, adoption, implementation, and maintenance of practices and results over time [12,14-17]. Our evaluation team will use PRISM as a guide by which to frame our evaluation efforts.

### Operation Partners

The TWP program evaluation will be facilitated by our partnerships with several national-level VA operations program offices. These partners include VA’s Diffusion of Excellence (DOE) office, which is leading the implementation efforts, VA’s National Podiatry Office, VA’s National Spinal Cord Injuries and Disorders Program Office, the VA Office of Nursing Services, and the Office of Connected Care – Telehealth Services. Each office has a vested interest in the success of the TWP program and a unique role in supporting its implementation.

### Design and Data Sources

Our team will use a mixed-methods assessment to achieve our evaluation aims. The evaluation plan includes a combination of site visits, semistructured interviews, surveys, and analysis of VA administrative data to evaluate the TWP implementation process and impact on outcomes from the perspective of multiple stakeholders.

### Setting

The TWP program is being implemented in 4 VA medical centers that are part of 1 VA regional network (and select CBOCs, engaged at each center’s discretion).

### Participants

Stakeholders include facility leadership, health care team members (ie, clinic managers, telehealth staff, and providers/wound care specialists), and veterans who receive TWP care from the participating VA medical centers and their CBOCs, as appropriate.

### Implementation Readiness

To prepare for the evaluation, our operations partners collected implementation readiness and preliminary process data. The preliminary process data were collected by DOE leadership at a TWP kick-off meeting hosted by the DOE and collaborating operational partners in the summer of 2019. These preliminary data were shared with the evaluation team. Kick-off meeting attendees (TWP site representatives) completed a readiness tool gauging their ability to implement TWP care.

Our evaluation team followed up with the collection of additional implementation readiness data via telephone with TWP clinical champions at each implementation site. Data collected from each site’s clinical champions included when the site began providing TeleWound care, the status of equipment (eg, 3-dimensional [3D] cameras), the training of staff, and the extent of the program, including which clinics and CBOCs were involved and what types of telehealth they were providing (eg, SFT, CVT, VVC). Because the implementation timelines differed across the 4 sites, we felt it was particularly important to document the date that each of these implementation milestones was reached to ensure that we will be able to accurately track implementation progress and program outcomes moving forward. In addition, we plan to conduct periodic check-ins via telephone calls with the implementation sites to monitor implementation progress.

### Planned Data Collection

#### Administrative Data

Data on patient-level and system-level outcomes and patient characteristics will be obtained from national VA administrative databases housed in the VA Corporate Data Warehouse (CDW). CDW data are refreshed nightly, allowing for real-time monitoring of patient-level health and utilization information. We will obtain the following patient characteristics from the CDW: demographics (age, gender, marital status, race, income), diagnosis, number of outpatient encounters, number of inpatient admissions, VA enrollment priority group, rurality of residence, and provider location and specialty. We will obtain the following utilization data on a monthly basis: number of veterans who had a TeleWound encounter, number of TeleWound encounters by clinic and location, type of telehealth visit, and visit diagnoses. To measure the impact of the TWP on patient travel cost and burden, we will assess travel costs to receive wound care. Travel costs will be estimated using the distance from veterans’ homes to the VA health care facilities where they received wound care and the Internal Revenue Service standard business reimbursement rates for travel by private automobile. Distances will be estimated based on veterans’ and facilities’ ZIP codes [18,19]. Table 1 identifies the outcomes related to each aim of the evaluation and their corresponding data sources.

**Table 1:** Evaluation outcomes and data sources.

Outcome				Data source
**Aim 1: stakeholder perspectives and reach of the program**				
	Implementation barriers and facilitators			Patient surveys/interviews, provider surveys/interviews, site visits
	Patient satisfaction			Patient surveys/interviews, site visits
	Provider satisfaction			Provider surveys/interviews, site visits
	TWP^a^ utilization: number and type of telehealth			CDW^b^
	Number of providers engaged			CDW
**Aim 2: clinical outcomes related to wound care**				
	**Hospitalizations following the initial TeleWound encounter**			
		Within 30 days	CDW	
		Within 90 days	CDW	
		Within 12 months	CDW	
	**Amputations following the initial TeleWound encounter**			
		Within 30 days	CDW	
		Within 90 days	CDW	
		Within 12 months	CDW	
**Aim 3: health care systems outcomes**				
	Mean number of miles traveled to receive wound care			CDW
	Mean travel cost			CDW for ZIP codes, federal reimbursement rates

^a^TWP: TeleWound Practice.

^b^CDW: Corporate Data Warehouse.

#### Veteran Survey

Surveys assessing veteran experiences with and perceptions of TeleWound care will be deployed in VA fiscal year 2020-2021, no earlier than 3 months after the facilities included in our evaluation have implemented the TWP. We will identify veterans who received TeleWound care using clinic stop codes and the TeleWound workload capture code (4-Character National Code defined as WCUC) recorded in the CDW.

Survey mailing processes will follow a modified version of the principles of tailored design [20]. We will first mail veterans an introductory letter providing a brief overview of the study and invitation to participate, along with a copy of the survey, a small appreciation gift (eg, refrigerator magnet), and a postage-paid return envelope. We will send reminders and a second survey to veterans who have not responded within 6 weeks of the first mailing. A thank-you letter will also be sent upon completion of the survey.

The veteran survey will follow the dimensions of the PRISM model and interests of our operational partners. This survey will collect data on the type of TeleWound services received, veteran perceptions of TeleWound care, quality of life, wound self-management, and demographics.

#### Veteran Semistructured Interviews

At the end of the veteran survey, participants will be asked to indicate whether they are willing to complete a semistructured telephone interview to provide more details about their experiences receiving TeleWound care. Those who agree will be invited to participate in the interview. If possible and as appropriate, we will select respondents that represent the 4 implementation sites and that have received different types of TeleWound care (ie, SFT, CVT, VVC).

We will conduct interviews with up to 25 veterans. All interviews will be one-on-one, semistructured, about 30 to 45 minutes in duration, and audio-recorded for verbatim transcription and subsequent analysis. The semistructured interview guide will further explore the topics covered in the survey, including experiences with TeleWound care, perceptions of TeleWound care, whether they received training or engaged in other preparation activities prior to their TeleWound visits, the impact of TeleWound care on their wound self-management, and suggestions for improvement.

#### VA Care Team Member Survey

We will also conduct an online survey with VA care team members involved in the provision of TWP care. We will work with our operations partners and site leadership to identify appropriate providers and staff to reach out to, which could include individuals who work in a range of areas (eg, spinal cord injury, nursing, podiatry, infectious disease, endocrinology, primary care).

The survey and sampling frame list will be managed through the VA-approved Research Electronic Data Capture (REDCap) system. Eligible VA care team members will be sent an email introducing the study and inviting them to participate and the link to the survey. To achieve an optimal response rate, an email reminder with the link to the survey will be sent 2 weeks later. A final email reminder will be sent 2 weeks after that. Email recruitment efforts will be augmented with instant messages (IMs) sent via interoffice IM.

Like the veteran surveys, care team member surveys will collect data on topics that map onto the PRISM model, as well as those identified as important by our operational partners. The topics addressed within the survey will include perceptions of and experiences with telehealth, perceptions of TeleWound training, barriers and facilitators to delivering TeleWound care, availability of equipment and other resources needed to deliver TeleWound care, suggestions for improvements, and demographics.

#### Key Stakeholder Semistructured Interviews

Key stakeholder interview procedures will follow those of the patient interviews detailed above. We will ask participants of the VA care team member survey to indicate whether they are willing to be contacted for a semistructured telephone interview at the end of the survey. Agreeable individuals will be invited to participate in an interview. We will also use snowball sampling to identify additional care team members and facility leadership to invite to participate, as appropriate.

We will conduct interviews with up to 20 key stakeholders. All interviews will be one-on-one, semistructured, and audio-recorded for verbatim transcription and subsequent analysis. Interviews will be approximately 30 minutes long. Semistructured interview guides will be used to ensure that comparable topics are covered across interviews.

Key stakeholder interviews will provide data to complement survey data, including information about implementation processes (including training and equipment), barriers and facilitators to TWP implementation, program reach and impact, and perceptions of the TWP and its implementation.

#### Site Visits

Site visits are a type of field-based data collection strategy in which one can observe, interact, and understand people and programs in their natural setting [21]. Our evaluation team will conduct site visits at 2 implementation sites (and their associated CBOCs, as appropriate). Site visits will provide us with an opportunity to observe the TWP from both the main facility and the rural CBOC sites to get a richer understanding of the process and workflow involved in using TWP. The site visit activities will be used to inform our team’s interpretation of the data collected from other sources (eg, interviews, surveys, administrative data).

During the site visits, our team will conduct additional key stakeholder interviews with individuals (eg, nurse managers, telehealth clinical technicians, licensed practical nurses, wound care specialists) at the selected main facility and their CBOCs using the interview guide described above. Field notes will also be taken to record observations of the TWP at the main facility and its CBOCs in real time. Flow maps of the TeleWound process will be developed based on our observations and input from interviews.

### Data Analysis

#### Quantitative Analyses (Survey and Administrative Data)

Survey and administrative data will be analyzed using descriptive and bivariate statistics, including means, medians, frequencies, standard deviations, chi-square tests, and Wilcoxon rank sum tests. Our evaluation team will compare the change in use of telehealth for wound care in participating sites before and after TWP implementation.

Specifically, we will compare the frequency and percentage of veterans who had wound care visits who used telehealth before and after TWP implementation using a chi-square test. Type of TeleWound care will be identified and responses will be compared across these modalities (ie, SFT, CVT, VVC). We will also look at the demographic characteristics of patients who received TeleWound care versus in-person care in a clinic to see if rural veterans were more likely to receive TeleWound services. The frequency and percentage of unique veterans with hospitalizations for wound-related complications within 30 days, 90 days, and 12 months following an initial wound encounter will be described and compared between veterans who received TeleWound care and veterans who received wound care in person. Frequency and percentage of unique veterans with amputations for wound-related complications within 30 days, 90 days, and 12 months following an initial wound encounter will also be analyzed. In addition, we will describe and compare the mean number of miles traveled to receive wound care and the mean travel costs over a 12-month period between veterans who received TeleWound care and veterans who received wound care in person. Finally, to examine uptake of TeleWound in each facility, we will identify the number of unique providers involved in TeleWound as well as the range of wounds treated (eg, pressure injuries, burns, chronic wounds). Analyses will include unadjusted and multiple regression models, which will assess the impact of the TWP on key outcomes (eg, hospitalizations, amputations, travel burden, costs).

#### Qualitative Analyses

Members of our evaluation team will use content analysis, including the constant comparative method, to identify and tabulate key themes emergent from the data [22]. Our coding approach will be both deductive and inductive and informed by our site visits. Coders will develop an initial code list a priori based on the components of the PRISM model, which encompasses a range of factors (as described above) that may influence the implementation of the TWP. Within the components of the model, coders will inductively develop additional codes and analyze the text for themes and patterns. Field notes recorded at the site visits will also be used to help identify these initial codes. Upon completion of deductive coding, a series of meetings will be held with members of the larger evaluation team to identify further themes as needed.

#### Data Triangulation

Our evaluation plan will use an explanatory sequential design, wherein quantitative data will be collected and analyzed first, followed by qualitative data. Once analyses are complete, members of our evaluation team will integrate qualitative and quantitative data using side-by-side comparisons of the qualitative data and joint displays, which include qualitative themes and selected dimensions from the quantitative data [23].

## Results

This program evaluation was funded in October 2019 as a Partnered Evaluation Initiative by the US Department of Veterans Affairs, Diffusion of Excellence Office, and Office of Research and Development, Health Services Research and Development Service, Quality Enhancement Research Initiative Program (PEC 19-310).

## Discussion

### Partnered Program Evaluation in a Learning Health Care System

Literature favoring the integration of telehealth into care provision continues to accumulate, suggesting that offering care virtually is effective and can improve important outcomes, such as access to health care [5,6,24-26]. Within this body of literature, evidence suggesting that delivering wound care using telehealth is feasible, improves health care outcomes, and reduces costs [27-29] is burgeoning. Using available evidence to inform health care is aligned with the goals of a large learning health care system such as VA and is illustrated by the TWP program, including the investment VA has made in establishing relationships between leadership, front-line clinical staff, and program evaluators to support its implementation.

The evaluation we describe here illustrates this strong partnership between our evaluation team and operations leadership. To date, DOE leadership has been a tremendous asset to our evaluation efforts and has also assumed responsibility for conducting the implementation of the TWP. One important aspect of implementation activities thus far, which was organized and run by the DOE, is holding regular calls with key participants from the sites and operational partners to monitor progress, identify problems, problem solve, and share solutions. Moreover, DOE leadership obtained and facilitated training on the 3D cameras, which some sites will be using to provide SFT TeleWound care. In addition, the DOE organized the TWP kick-off meeting, wherein key informants and operations partner representatives reviewed plans and shared early experiences.

Our evaluation team has also been able to leverage these operations-driven implementation activities as the foundation of our TWP evaluation. The information and experiences detailed through these important early implementation milestones have helped to inform the content of our data collection instruments and plans for timing of data collection efforts. Similarly, our early evaluation findings have and will continue to be fed back to DOE and other operations leadership, allowing them to enhance the effectiveness of their implementation plans in real time. These synergistic implementation and evaluation activities underscore the importance of partnerships between operations leadership, key stakeholders, and program evaluators to the success of program implementation and evaluation alike.

### Limitations

Our evaluation team is not facilitating the implementation of the TWP program; however, we are and will continue to provide feedback to the DOE about implementation progress and barriers as our evaluation activities move forward. Because of the pragmatic nature of implementation studies, there is less control of processes and structures during an implementation project than other more controlled types of projects, but this also increases ecological validity. Our strategies for primary data collection are subject to biases, including recall bias and social desirability.

### Conclusions

Regular communication between implementation sites, DOE leadership facilitating the implementation, and the evaluation team is key to tracking implementation progress, lessons learned, and barriers and facilitators to the success of the TWP. An important consideration for our evaluation work is that TWP implementation is occurring in a dynamic environment and as such, new equipment, changing staff and leadership, and varying facility and staff priorities and demands may arise during the implementation period. Changes such as these may make implementation challenging but highlight the ever-present importance of keeping the goals of TWP and the needs of veterans at the forefront of efforts to implement and evaluate activities.

The efforts to date on this project have demonstrated how critical early investments in infrastructure are to the success of TWP program implementation. The TWP program has unique needs and requirements to be addressed before the program can be implemented, and in turn, the implementation process and subsequent outcomes can be evaluated. The evaluation plans we detail will inform efforts moving forward and will be integral to the facilitation of TWP throughout VA.

## Abbreviations

3D: 3-dimensional
CBOC: community-based outpatient clinic
CDW: Corporate Data Warehouse
CVT: clinical video telehealth
DOE: Diffusion of Excellence
IM: instant message
PRISM: Practical, Robust Implementation and Sustainability Model
RE-AIM: Reach, Effectiveness, Adoption, Implementation, Maintenance
SFT: store-and-forward
TWP: TeleWound Practice
VA: Veterans Health Administration
VVC: Veterans Health Administration Video Connect
